# Quantitative trait loci mapping of panicle traits in rice

**DOI:** 10.22099/mbrc.2019.31550.1366

**Published:** 2019-03

**Authors:** Baoyan Jia, Xinhua Zhao, Yang Qin, Muhammad Irfan, Tae-heon Kim, Bolun Wang, Shu Wang, Jae Keun Sohn

**Affiliations:** 1 Department of Agronomy, Shenyang Agricultural University, 110161, Shenyang China; 2 Department of Agronomy, Kyungpook National University, 702-701, Daegu, Republic of Korea; 3 Department of Biotechnology, University of Sargodha, Sargodha 40100, Pakistan

**Keywords:** QTLs, Rice, Panicle traits, SSR, RILs

## Abstract

In this study 90 individuals of recombinant inbred lines (RILs) were developed by crossing subspecies of *japonica* rice cultivar, ‘Nagdong’ and an *indica* type cultivar, ‘Cheongcheong’. These individuals were used to identify the quantitative trait loci of panicle traits using SSR markers. A genetic linkage map was constructed using one hundred fifty four simple sequence repeat (SSR) primers covering distance of 1973.6 cM of the whole genome with mean distance of 13.9 cM among markers. QTLs were mapped using composite interval mapping method, nineteen QTLs were recognized for the panicle traits on chromosomes 4, 5, 6, 8, 10, 11, 12 with individual QTL explained 8.8% to 37.9% of phenotypic variation. Two pleiotropic effects loci were found on chromosomes 4 and 6. These QTLs affecting leaf traits, panicle traits and panicle branch traits would be beneficial to high-yield rice improvement.

## INTRODUCTION

In rice the panicle is the top organ, an important component of the canopy. Average spikelet number per panicle, number of panicle per unit area and grain weight determine spikelet yield [1]. A large number of spikelets can be reached by increasing the number of panicle or the number of spikelets per panicle. An increase in number of panicle per plant by increasing tillers may result in large sink capacity. However, excess tillering may result in high rate of tiller abortion, small size of panicle, poor filling of grain, and eventually reduced grain yield in rice. A new variety of rice has been developed by IRRI which has large spikelets number per panicle and a less panicles number per plant [2]. The characters of rice panicles, such as percent seed set, number of filled grain per plant and length of panicle are key traits to improve yield [3]. Panicle characters such as number of the primary and second branches influence spikelet number per panicle, number of full grain and length of panicle accounts for a major contribution to yield as compared to panicle number or kilo-grain weight [4,5]. Panicle traits such as spikelets per panicle and primary branch number per panicle are inherited quantitively [6-9]. The main aim of this study was the identification and mapping of quantitative trait locus of panicle traits in rice. 

## MATERIALS AND METHODS

**Plant materials and mapping population** **: **The detailed description of population used for mapping were mentioned in our earlier report [10]. 

**Phenotype data collection** **: **Panicle traits studied in this study were measured during the mature stage and included panicle number per plant (PNPP), panicle length (PL), number of primary branches per panicle (NPB), number of second branches per panicle (NSB), second branches per primary branch (SBPPB), number of spikelets per panicle (NSPP), full grain number (FGN), grain density (GD). For sampling 12 representative plants in the middle of plot were selected, and main stem leaves and panicles were selected for trait measurement and further analysis.

**DNA extraction and SSR** **-PCR amplification: **Genomic DNA was extracted from fresh-frozen leaves using CTAB method [11] and SSR-PCR amplification was done in detail as described in our earlier reports [10]. 

**Linkage map construction and QTLs identification** **: **Polymorphisms among the two parents Cheongcheong and Nagdong were distinguished with 580 SSR markers distributed randomly on the 12 chromosomes of rice and the polymorphic markers were then used to detect polymorphisms of RILs. Mapmaker/EXP Version 3.0 was used to create the linkage map. QTL identification procedure was described in our previous reports [10]. 

## RESULTS

**Figure 1:**
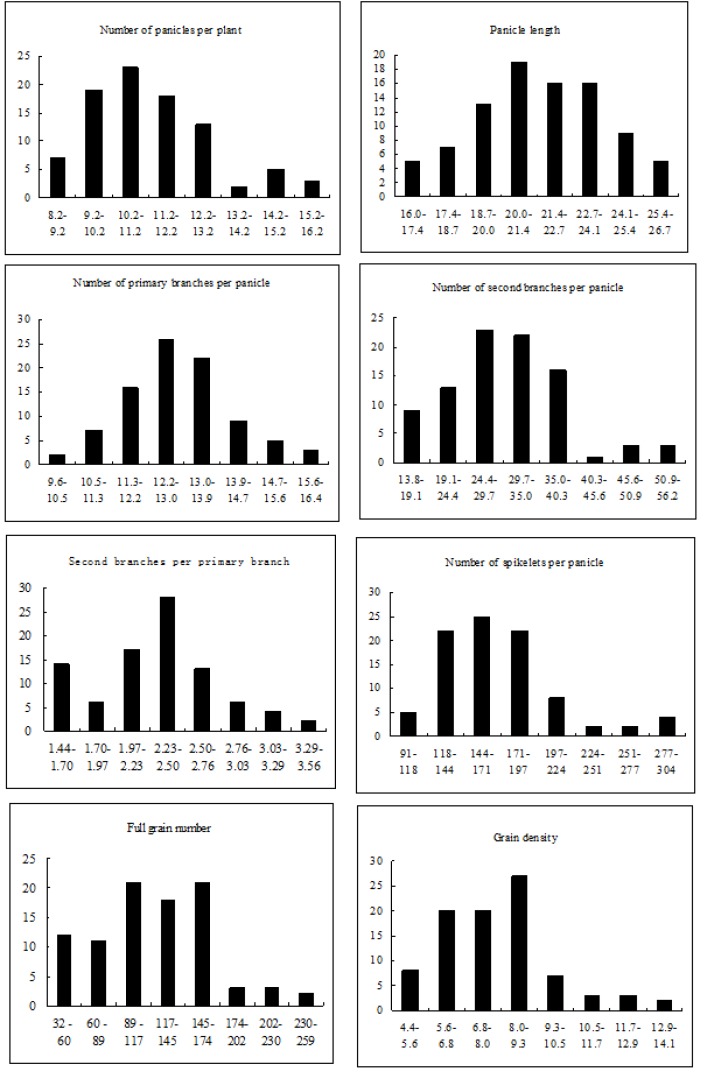
Distribution of panicle traits in the RILs population

The phenotypic differences of two parents and the population of RILs for PNPP, PL, NPB, NSB, SBPPB, NSPP, FGN and GD were presented in Table 1. The data showed that differences of parents in PNPP, PL, NSB, SBPPB and GD were significant at 5% level or 1% level. Leaf traits showed continuous distribution and transgressive segregation among RILs population (Fig.1) and except NSPP and GD, other traits’ skewness and kurtosis were less than 1.0 indicating the quantitative inheritance of panicle traits in the population which suggested its suitability for QTL analysis.

**Table 1:** Panicle performance of the RILs population and its parents

**Traits**	**Parents**				**RILs**		
	**Cheongcheong**	**Nagdong**	***t*** ** value**	**Mean**	**Range**	**Skew**	**Kurtosis**
PNPP	11.15	12.85	3.25^*^	11.27	8.17-16.25	0.847	0.661
PL	23.23	21.87	2.63^*^	21.51	16.02-26.73	-0.090	-0.414
NPB	11.91	12.20	0.50	12.95	9.60-16.40	0.149	0.142
NSB	22.82	26.28	4.27^**^	30.11	13.80-56.20	0.695	0.796
SBPPB	1.91	2.16	2.78^*^	2.30	1.44-3.56	0.127	-0.012
NSPP	144.46	144.10	1.14	169.22	91.40-304.20	1.143	1.699
FGN	127.71	136.47	1.04	122.13	32.38-259.26	0.346	0.377
GD	6.17	6.60	3.72^**^	7.90	4.39-14.13	0.904	1.271

**Figure 2:**
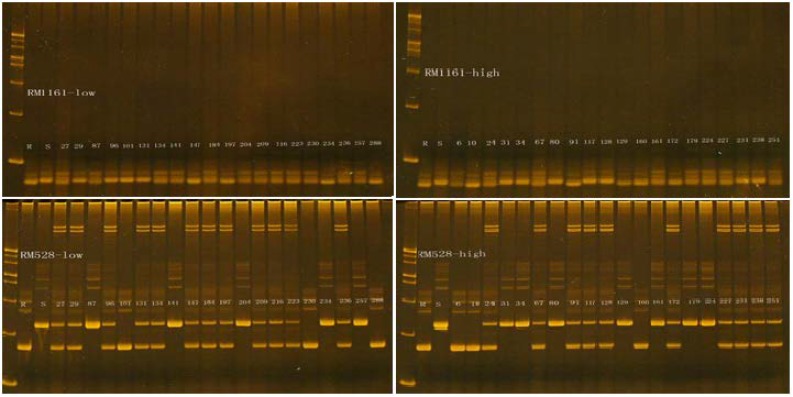
Comparison of band produced by DNA makers related to the NSPP using F_2_ derived from the cross ‘Reimei/Shendao4’. ‘R’ represent Reimei, ‘S’ represent Shendao4

**Figure 3:**
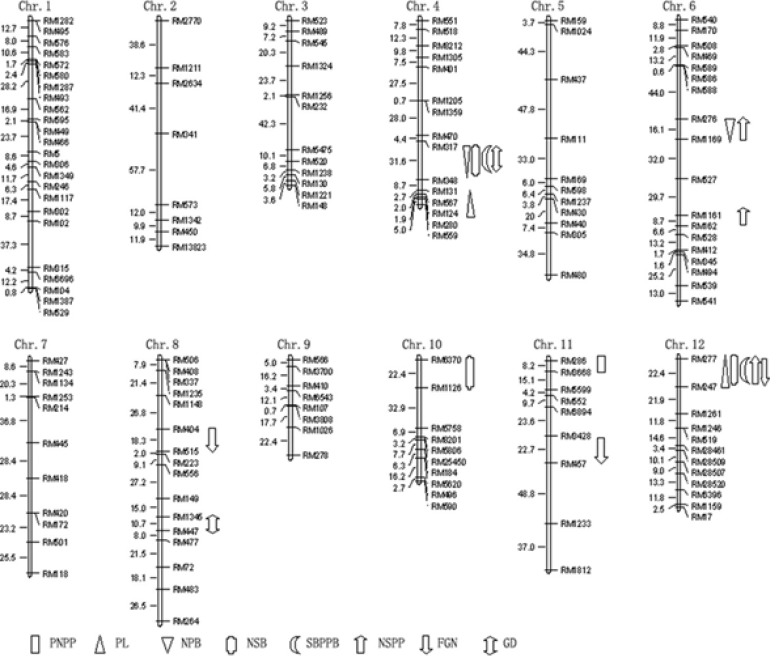
QTLs for panicle traits were identified in the RILs population

Genotyping of the population was done by SSR markers as shown in the Figure 2 and data was collected for further QTL identification. By the composite interval mapping method, the significant QTLs were identified for the 8 panicle traits are mentioned in Table 2. Nineteen QTLs were detected for the panicle traits on 4,5,6,8,10,11,12 chromosomes (Fig. 3) with individual QTL explained 8.8% to 37.9% of phenotypic variation. For PNPP, one QTL was identified on chromosome 11, accounting for 21.6% of the phenotypic variation. The Nagdong alleles contributed to increase panicle number per plant. Two QTLs for PL were recognized on 4 and 12 chromosome, having phenotypic variation of 35.4%. QTLs for NPB, which explained 47.5% of phenotypic variation, were detected on chromosomes 4, 5 and 6. The Nagdong alleles were associated with increasing primary number of branches per panicle at *qnpb4.1* and *qnpb6.1*. For NSB, Three QTLs were identified on chromosomes 4, 10 and 12, and these QTLs explained phenotypic variation of 60.7%. The Nagdong alleles were associated with increasing number of second branches per panicle at *qnsb4.1* and *qnsb12.1.*

**Table 2:** QTLs for panicle traits detected using a recombinant inbred lines (RILs**) **population

**Traits**	**QTL**	**Chr.**	Marker interval	**Increase allele**	**LOD**	***R***^2^	**Addictive effect**
NPP	*qpnpp11.1*	11	RM286-RM3668	Nagdong	4.95	21.6	0.843
PL	*qpl4.1*	4	RM131-RM124	Cheongcheong	3.88	13.6	-0.934
	*qpl12.1*	12	RM277-RM247	Nagdong	4.38	21.8	1.138
NPB	*qnpb4.1*	4	RM317-RM348	Nagdong	3.05	18.9	0.602
	*qnpb5.1*	5	RM159-RM1024	Cheongcheong	3.92	13.5	-0.505
	*qnpb6.1*	6	RM276-RM1169	Nagdong	3.72	15.1	0.587
NSB	*qnsb4.1*	4	RM317-RM348	Nagdong	3.48	23.6	4.353
	*qnsb10.1*	10	RM6370-RM1126	Cheongcheong	3.03	8.8	-2.662
	*qnsb12.1*	12	RM277-RM247	Nagdong	5.65	28.3	4.863
SBPPB	*qsbppb4.1*	4	RM317-RM348	Nagdong	4.73	37.9	0.304
	*qsbppb12.1*	12	RM277-RM247	Nagdong	5.26	26.1	0.256
NSPP	*qnspp.6.1*	6	RM276-RM1169	Nagdong	3.33	10.0	15.032
	*qnspp6.2*	6	RM1161-RM528	Cheongcheong	3.48	12.3	-17.757
	*qnspp12.1*	12	RM277-RM247	Nagdong	5.28	25.0	21.862
FGN	*qfgn8.1*	8	RM404-RM515	Cheongcheong	2.81	15.3	-18.988
	*qfgn11.1*	11	RM3428-RM457	Nagdong	2.93	25.3	23.782
	*qfgn12.1*	12	RM277-RM247	Nagdong	4.36	22.7	22.756
GD	*qgd4.1*	4	RM317-RM348	Nagdong	4.30	23.8	0.931
	*qgd8.1*	8	RM1345-RM447	Cheongcheong	4.59	16.3	-1.016

Two QTLs for SBPPB were identified on chromosomes 4 and 12, which explained 64.0% of phenotypic variation. Nagdong alleles increased Second branches per primary branch at these QTLs. For NSPP, four QTLs were detected on 6 and 12 chromosome having phenotypic variance of 47.3%. Three QTLs for FGN were known on 8, 11 and 12 chromosome, which explained 63.7% of phenotypic variation. The Nagdong alleles were contributed to increasing full grain number at *qfgn11.1* and* qfgn12.1. *For GD, two QTLs were detected on 4 and 8 chromosome having phenotypic variance of 41.1%. One pleiotropic effects locus was identified on 4 chromosome with marker interval of RM317-RM348 for *qnpb4.1*, *qnsb4.1, qssppb4.1 *and* qgd4.* One pleiotropic effects locus was identified on chromosome 6 at the interval RM277-RM247 for *qpl12.1, qnsb12.1, qsspb12.1, qnspp12.1, qfgn12.1*.

## DISCUSSION

Rice breeding programs have focused on yield improvement. The average spikelet number per panicle, panicle number per unit area and grain number of panicles per unit area and grain weight determined spikelet yield. Among them, the number of panicle per unit area shows a low heritability and is largely influenced by environmental factors [12]. Malaysian rice varieties had panicle length in rage of 24.4-30.0, 8.0-18.0 number of panicles per plant and 64.0-321.0 number of grains per panicle [13]. Panicle length ranging from 19.5-32.17 and grain number in panicle having variation of 75-324.7 has been reported in advanced backcross lines of rice [14]. Rahman *et al.,* [15] analyzed panicle number in range of 7.2–18.8, and number of spikelet per panicle in range of 151-239 in F3 population of hybrid rice.

A total of 19 QTLs associated with panicle traits were identified on chromosome 4, 6, 8, 10, 11 and 12. These QTLs explained 8.8%-37.9% of phenotypic variation, and distribute 10 regions on the chromosomes. Among these regions, RM277-RM247 increased PL, NSB, SBPPB, NSPP and GD with the Nagdong allele. All of QTLs associated with these traits explained more than 20% of phenotypic variation respectively. This indicates that it was important for the panicle traits, and could be further studied for improved panicle type and obtain high grain yield. In the present study two QTLs were identified on chromosome number 4 and 12 which were almost in agreement with previous reports. Liu *et al.,* [16] reported four QTLs for panicle length on chromosome number 4,6 and 9 in RIL population of rice. Vemireddy *et al.,* [17] reported two QTLs for panicle length on chromosome 2 and 6 having minor effects in Basmati rice population. Zhang *et al.,* [18] identified two QTLs for panicle length in backcross population of rice on chromosome 4 and 6. In another study, three QTLs for panicle length were identified on chromosome number 1,3,9 in RIL population of rice [19]. Two single locus QTLs of panicle length were detected on chromosome number 4 and 6 in F_2_ and F_3_ population of hybrid rice [15].

RM317-RM348 increased NPB, NSB, SBPPB and GD with the Nagdong allele, and *qnsb4.1*, *qsppb4.1* and *qgd4.1* explained 23.6%, 37.9% and 23.8% of phenotypic variation respectively. Because there was long distance between two markers, so we can not sure if these were main-effect QTLs. Five QTLs for NPB and three QTLs for NSB were identified having explained variance of 5.6-19.3% and 4.9-26.4% respectively [12]. 

RM1161-RM162 increased NSPP with the Cheongcheong allele, and *qnspp6.2* explained 12.3% of phenotypic variation. In this study 3 QTLs were mapped on chromosome 6 and 12 which was consistent with previous studies. Rabiei *et al.,* [20] mapped one QTL for each number of spikelet per panicle and panicle length on chromosome 12 and 1 using F_2:4_ population of rice respectively. Sabouri *et al.,* [21] detected seven QTLs for number of spikelet per panicle on chromosome number 2,3,4,5,12 in F_2_ population of rice. One QTL for number of spikelet per panicle was identified on chromosome 6 in both F_2_ and F_3_ population of hybrid rice [15].
